# Recovery of platinum(0) through the reduction of platinum ions by hydrogenase-displaying yeast

**DOI:** 10.1186/s13568-016-0262-4

**Published:** 2016-10-04

**Authors:** Rio Ito, Kouichi Kuroda, Haruka Hashimoto, Mitsuyoshi Ueda

**Affiliations:** Division of Applied Life Sciences, Graduate School of Agriculture, Kyoto University, Sakyo-ku, Kyoto, 606-8502 Japan

**Keywords:** Platinum, Metal reduction, Metal recovery, Hydrogenase, Cell membrane display, *Saccharomyces cerevisiae*

## Abstract

Biological technologies for recycling rare metals, which are essential for high-tech products, have attracted much attention because they could prove to be more environmentally friendly and energy-saving than other methods. We have developed biological recycling technologies by cell surface engineering for the selective recovery of toxic heavy metal ions and rare metal ions from aqueous wastes. In this study, we aimed to construct a unique biological technique to recover rare metals ‘in solid’ form by reducing rare metal ions, leading to a practical next-generation recovery system. Sulfate-reducing bacteria (SRB) can reduce Pt(II) to Pt(0), and hydrogenases of SRB contribute to the reduction. Therefore, we constructed yeasts displaying their hydrogenases on the ‘cell membrane’, and reduction experiments were performed under anaerobic conditions without any electron donors. As a result, hydrogenase-displaying yeasts produced black precipitates in PtCl_4_^2−^ solution. Based on X-ray photoelectron spectroscopy (XPS) and transmission electron microscopy (TEM) observations, the constructed yeasts were found to successfully produce the precipitates of Pt(0) through the reduction of Pt(II). Interestingly, the precipitates of Pt(0) were formed as nanoparticles, suitable for industrial usage.

## Introduction

Rare metals are essential for high-tech products such as cell phones and automobiles, leading to ever-growing demands today. However, a stable supply of rare metals is difficult on a global scale because rare metals are expensive and localized, and their abundances are extremely low. Therefore, technologies for recovering rare metals from waste products, industrial wastewater, and sea are required.

Recently, various physical, chemical, and biological methods have been studied to prepare metallic forms for the recycling of rare metals (Du et al. 2008; Fredrickson et al. 2008; Cueto et al. 2011). However, physical and chemical methods have some disadvantages. For example, they involve toxic solvents and consume high energy. On the other hand, biological methods can produce metallic forms in a more environmentally friendly and energy-saving manner than other methods, because they only require microbes as the basic constituent. Therefore, biological methods have currently attracted much attention, and various microbes that produce metallic forms have been screened on the earth (Klaus-Joerger et al. 2001; Konishi et al. 2006).

Platinum, especially, is a rare precious metal used in catalytic converters or fuel cells. However, there are growing concerns about platinum exhaustion by 2050 because its crustal abundance is extremely low. Additionally, the Republic of South Africa accounts for about 70 % of platinum production. Therefore, developing efficient technologies to accumulate and recycle platinum is desired to ensure a stable and continuous supply of platinum.

It has been reported that sulfate-reducing bacteria (SRB) contribute to the bioremediation of toxic metal ions such as chromate or uranium by reducing them (Michel et al. 2001; Payne et al. 2002). SRB are anaerobic prokaryotes found ubiquitously in nature and many studies of SRB have been conducted on the genus *Desulfovibrio*. It became clear afterwards that SRB can reduce tetrachloroplatinate (PtCl_4_^2−^) in aqueous solutions to elemental platinum [Pt(0)] and that hydrogenases on the cell membrane of SRB contribute to the reduction reaction (Riddin et al. 2009).

We have developed biological recycling technologies using cell surface engineering for the selective recovery of rare metals and toxic heavy metals causing environmental pollution (Ueda and Tanaka 2000; Kuroda and Ueda 2010, 2011; Nishitani et al. 2010; Kuroda et al. 2012). In cell surface engineering, any metal-binding proteins/peptides can be displayed on the yeast cell surface with maintenance of their activities. Furthermore, ‘cell membrane’ display of target proteins (Hara et al. 2012) has been achieved by fusing the target proteins to plasma membrane proteins such as Yps1p (Gagnon-Arsenault et al. 2006). The adsorbed metal ions can be easily recovered from yeast cells, because they are adsorbed on the yeast cell surface and a metal recovery process does not need breaking down the yeast cells. Therefore, we can repeatedly use the surface-engineered yeast cells as bioadsorbents in the next reaction.

In this study, we attempted to construct yeasts that display hydrogenase on their ‘cell membrane’ and use the constructed yeasts to recover platinum in ‘solid form’ by reducing Pt(II) to Pt(0). Yeast cells grow faster than SRB and are easily handled, because of the simple medium required to culture yeast cells and their safe biological features as compared to those of SRB. Therefore, it is likely that hydrogenase-displaying yeasts could recover platinum more effectively than SRB. In addition, platinum is mainly used as nanoparticles in catalytic converters of automobiles. Our method to recover platinum as nanoparticles, not simple solid forms, using hydrogenase-displaying yeasts could be useful as a next-generation technology for efficient metal recovery.

## Materials and methods

### Strains and media

*Escherichia coli* DH5α [F^−^, $\phi$80d*lacZΔ*M15, *Δ*(*lac*ZYA-*arg*F)U169, *hsd*R17($r_{K}^{ - }$, $m_{K}^{ + }$), *rec*A1, *end*A1, *deo*R, *thi*-1, *sup*E44, *gyr*A96, *rel*A1, *λ* ^−^] was used as a host for recombinant DNA manipulation. *Saccharomyces cerevisiae* W303-1A (*MAT* **a**, *ura3*-*1, leu2*-*3/112, trp1*-*1, his3*-*11/15, ade2*-*1, can1*-*100*) was used as the host for the display of proteins on the cell membrane. *Desulfovibrio vulgaris* Hildenborough (ATCC 29579) and *Desulfovibrio desulfuricans* (ATCC 27774) were used to amplify the hydrogenase genes from their genomic DNAs. *E. coli* was grown in Luria–Bertani medium [1 % (w/v) tryptone, 0.5 % yeast extract, and 0.5 % sodium chloride] containing 100 μg/mL ampicillin. Yeast cells were precultivated anaerobically in synthetic dextrose medium (SDC + AHLU) [0.67 % (w/v) yeast nitrogen base without amino acids, 2 % glucose, 0.5 % casamino acids, 0.002 % adenine sulfate dehydrate, 0.002 % l-histidine, 0.003 % l-leucine, and 0.002 % l-uracil] bubbled with N_2_ gas, and then cultivated in the same medium.

### Construction of plasmids for cell membrane display

All primers used in the plasmid construction are listed in Table 1. The genomic DNAs were extracted from *D. vulgaris* Hildenborough and *D. desulfuricans* cell cultures using Blood & Cell Culture DNA Kit (Qiagen, Hilden, Germany). The DNA fragment encoding Hyn B hydrogenase (Gene-ID: 2793337) was amplified from *D. vulgaris* Hildenborough genomic DNA by PCR using primers Hyn B FW and Hyn B RV. In addition, the DNA fragment encoding Ni-Hyd hydrogenase (Gene-ID: 7284719) was amplified from *D. desulfurican* genomic DNA by PCR using primers Ni-Hyd FW and Ni-Hyd RV. The amplified DNA fragments Hyn B and Ni-Hyd were inserted into *Bam*HI section of the pYS0 plasmid (Hara et al. 2012) for the Yps1p-based display on yeast cell membrane by using the In-Fusion HD Cloning Kit (Clontech, CA, USA). The resulting plasmids were named pYS-Hyn B and pYS-Ni-Hyd, respectively. The plasmid pYS0 for the cell membrane display of the FLAG-tag was used as a negative control for the platinum reduction experiments.

**Table 1:** Primers used in this study

Primer name	Sequence	Application
Hyn B FW	5′-TTGCTCGTTTCTGCCATGCGATTCTCAGTCGGTCTTGGC-3′	Cloning of Hyn B
Hyn B RV	5′-GTCATCCTTGTAATCGCTTTCGTAGAACGGGCTCTTTTCG-3′	Cloning of Hyn B
Ni–Hyd FW	5′-TTGCTCGTTTCTGCCATGAGCCAAGTCACTAAAACGCCCC-3′	Cloning of Ni–Hyd
Ni–Hyd RV	5′-GTCATCCTTGTAATCCAGAACCTTGTAGTCATGCACTTCGTTG-3′	Cloning of Ni–Hyd

### Transformation of *S. cerevisiae*

*Saccharomyces cerevisiae* W303-1A was transformed with the constructed plasmids using the Frozen-EZ Yeast Transformation II kit (Zymo Research, Irvine, CA, USA). Transformed cells were isolated by incubation on SDC + AHLU agar plates at 30 °C for 48 h. All constructed strains are listed in Table 2.

**Table 2:** List of constructed yeast strains

Strain name	Feature
Hyn B	Displaying Hyn B on the cell membrane
Ni–Hyd	Displaying Ni–Hyd on the cell membrane
control	Displaying only FLAG tag as a negative control for platinum reduction

### Immunofluorescence microscopy

To confirm the display of hydrogenase on yeast cell membrane, immunofluorescent labeling of cells with or without cell wall digestion was performed using the antibody against the FLAG epitope tag as described previously (Nishitani et al. 2010). Yeast cells were cultivated in SDC + AHLU medium at 30 °C for 12 h. Yeast cells were collected from 2.0 mL of yeast suspension after centrifugation at 2000×*g* for 5 min at 4 °C, washed with Tris–EDTA buffer (pH 8.0), and suspended in 1 mL of cell-wall-digesting enzyme solution [0.1 M Mcllvain buffer (pH 6.0) containing 400 U/mL westase (Takara Bio Inc., Shiga, Japan) and 0.5 M sodium tartrate]. Cell wall digestion was performed at 30 °C for 16 h with reciprocal shaking. Cells were incubated in phosphate-buffered saline (PBS, pH 7.4) containing 1 % bovine serum albumin for 30 min prior to immunostaining. Mouse monoclonal anti-FLAG M2 antibody (Sigma, St. Louis, MO, USA) was used as the primary antibody at a dilution of 1:300. A mixture of cells and the antibody was incubated using a rotator for 1.5 h at room temperature. Cells were then washed with PBS (pH 7.4). Alexa Fluor 488-conjugated goat anti-mouse IgG antibody (Invitrogen, Carlsbad, CA, USA) diluted at 1:300 was then incubated with the cells using a rotator for 1.5 h at room temperature. After washing with PBS (pH 7.4), cells were suspended in 30 μL of PBS (pH 7.4) and observed using an inverted microscope IX71 (Olympus, Tokyo, Japan) through a U-MNIBA2 mirror unit with a BP470-490 excitation filter, PM505 dichroic mirror, and BA510-550 emission filter (Olympus). Live images were obtained using the Aqua-Cosmos 2.0 software (Hamamatsu Photonics, Shizuoka, Japan) controlling a digital charge-coupled device camera (C4742-95-12ER, Hamamatsu Photonics).

### Pt(II) reduction experiment

Prior to Pt(II) reduction, hydrogenase-displaying yeasts and control yeast were precultivated anaerobically in SDC + AHLU medium bubbled with N_2_ gas at 30 °C for 24 h. The cells were collected after centrifugation at 3000×*g* for 5 min at room temperature and incubated in PBS (pH7.4) containing 100 μM PtCl_4_^2−^ with an optical density of 20 at 600 nm. The reduction reaction was anaerobically performed at 37 °C for 72 h with shaking. After the incubation, cells and black precipitates were separated from the reaction solution by centrifugation at 3000×*g* for 10 min at room temperature. Then, the residual platinum ions in the supernatant were quantified by inductively coupled plasma mass spectrometry (ICP-MS; Agilent 7500cx, Agilent Technologies, Santa Clara, CA, USA).

### X-ray photoelectron spectroscopy and transmission electron microscopy (TEM)

After Pt(II) reduction, black precipitates and cells were collected from cell suspensions by centrifugation at 3000×*g* for 10 min at room temperature, and then lyophilized. Quantera SXM (Physical Electronics, Chanhassen, USA) was used for X-ray photoelectron spectroscopy (XPS) measurements using Kα lines of Al (100 μm, 24.8 W, 15 keV) as an X-ray source. The pass energy was 112.0 eV, and the step size was 0.100 eV.

Transmission electron microscopy (TEM) using a 200 kV TEM instrument (JEM-2010F, JEOL, Tokyo, Japan) was employed to investigate the microstructure of the precipitates.

## Results

### Construction of hydrogenase-displaying yeasts

**Fig. 1:**
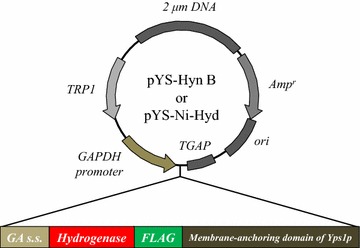
Plasmids for cell membrane display of hydrogenases. *GAPDH* glyceraldehyde-3-phosphate dehydrogenase, *GA s.s.* glucoamylase signal sequence from *Rhizopus oryzae*. Hydrogenase genes (*Hyn B* from *D. vulgaris* or *Ni*-*Hyd* from *D. desulfuricans*) were inserted between *GA s.s.* and *FLAG* of pYS0

**Fig. 2:**
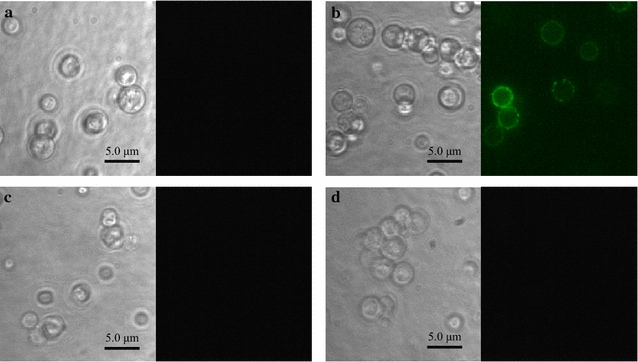
Immunofluorescent labeling of constructed yeasts. Immunofluorescent labeling was performed by using anti-FLAG antibody and Alexa Fluor 488 goat anti-mouse IgG antibody. **a** Hyn B-displaying yeast, **b** Hyn B-displaying yeast after cell wall digestion by westase, **c** wild-type yeast, and **d** wild-type yeast after cell wall digestion by westase. Phase contrast micrographs (*left*) and Immunofluorescence micrographs (*right*)

Display of hydrogenases on yeast cell membrane was attempted to mimic SRB that produce hydrogenases on their cell membrane for reduction activity. The constructed multicopy plasmids for cell membrane display of Hyn B from *D. vulgaris* and Ni-Hyd from *D. desulfuricans* (pYS-Hyn B and pYS-Ni-Hyd) contain a glucoamylase secretion signal (*GA s.s.*), hydrogenase genes (*Hyn B* or *Ni*-*Hyd*), a FLAG-tag, and a cell-membrane-anchoring domain of Yps1p (Fig. 1). To confirm the cell membrane display of the hydrogenases, immunofluorescent labeling of the transformed yeasts and wild-type yeast was performed with or without cell wall digestion, which improves the accessibility of the antibody to the membrane-displayed proteins (Fig. 2). The transformed yeasts after cell wall digestion showed a green fluorescence derived from fluorescent antibodies binding to the cell membrane, while other cells showed no fluorescence. The result indicates that transformed yeasts successfully displayed hydrogenases on their cell membrane.

### Platinum reduction by hydrogenase-displaying yeasts

**Fig. 3:**
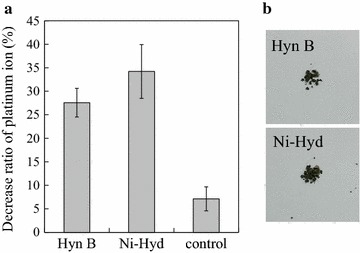
Platinum reduction by hydrogenase-displaying yeasts. **a** The ratio of platinum decrease in reaction solutions after the reduction experiment was calculated by comparing the platinum concentrations in the supernatant before and after the reduction. Data represent the mean ± SD of three independent experiments. **b** Microscopic observation of black precipitates produced after the reduction experiment

To evaluate the platinum-reduction abilities of the constructed hydrogenase-displaying yeasts, platinum reduction experiment was performed with the hydrogenase-displaying yeasts and control yeast under anaerobic conditions. The platinum concentration in the supernatant solution before and after the reduction was measured by using ICP-MS as described in “Materials and methods” section. Then, the decrease ratio of platinum ions in the reaction solutions after the reduction experiment was calculated (Fig. 3a). Two types of hydrogenase-displaying yeasts decreased more PtCl_4_^2−^ in the reaction solutions than the control yeast. In addition, after the reduction experiment, black precipitates were produced by hydrogenase-displaying yeasts (Fig. 3b). These results suggest that black precipitates produced by hydrogenase-displaying yeasts contained platinum(0).

### Analysis of precipitates produced by hydrogenase-displaying yeasts

**Fig. 4:**
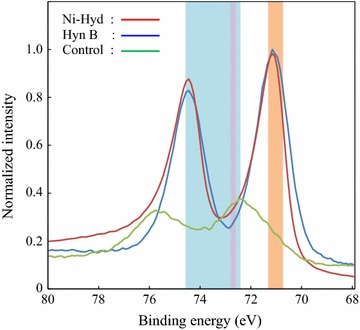
XPS spectra of black precipitates. The black precipitates produced after the reduction were analyzed by X-ray photoelectron spectroscopy (XPS). Ni-Hyd, black precipitates produced by Ni-Hyd-displaying yeast (*red line*); Hyn B, black precipitates produced by Hyn B-displaying yeast (*blue line*); Control, precipitates produced by control yeast (*green line*). Binding energies of 70.7–71.3 eV (*orange*), 72.6–72.8 eV (*pink*), and 72.4–74.6 eV (*light blue*) correspond to Pt(0), Pt(OH)_2_, and PtO, respectively

It was unclear whether the produced black precipitates contained other platinum compounds as well as elemental platinum [Pt(0)]. Therefore, to reveal the chemical form of the platinum included in the black precipitates, the precipitates were analyzed by XPS that is used for examination of the chemical-bonding state and the valence of the element (Fig. 4). In the black precipitates produced by hydrogenase-displaying yeasts, Pt(0) peak (binding energy: 70.7–71.3 eV) was detected. In contrast, Pt(0) peak was not detected in the precipitates produced by control yeast. Therefore, the result indicates that black precipitates produced by hydrogenase-displaying yeasts contained Pt(0).

**Fig. 5:**
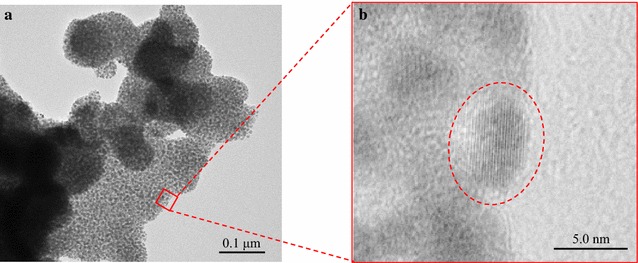
TEM images of black precipitates. Transmission electron microscopy (TEM) images of the black precipitates produced by Hyn B-displaying yeast (**a**) and its magnified image (**b**)

The black precipitates were observed by TEM to confirm the configuration of Pt(0) in the black precipitates (Fig. 5). The TEM image indicates that Pt(0) in the black precipitates existed as an aggregated nanoparticle. Furthermore, it was found that these nanoparticles had a crystalline structure because lattice fringes were present in the platinum nanoparticles (Fig. 5b, red dot circle).

## Discussion

We constructed yeasts displaying hydrogenase on their cell membrane and achieved the recovery of Pt(II) as nanoparticles using the constructed yeasts. So far, the concentration recovery of metal ions from aqueous solutions has been performed by the surface-engineered yeasts displaying the proteins/peptides that bind metal ions (Kuroda and Ueda 2010, 2011). In addition to this strategy, recovery of metals in a solid state from an aqueous solution is a remarkable strategy for practical use, in terms of the usability of the recovered metals. Especially, platinum is mainly used as nanoparticles in industries such as those producing catalytic converters of automobiles. In the case of platinum recovery as ions, an additional process for reducing the ions to nanoparticles is necessary. Therefore, our biological technique used to recover Pt(II) as nanoparticles can save cost and time, and is ecological because any electron donors are unnecessary in this system.

According to the result of XPS analysis (Fig. 4), the black precipitates produced by hydrogenase-displaying yeasts included Pt(0) with binding energies of 70.7–71.3 eV, suggesting that the hydrogenases displayed on yeast cell membrane contributed to the Pt(II) reduction to Pt(0). In addition, Pt(0) produced by hydrogenase-displaying yeasts formed nanoparticles with lattice fringes and the size of the platinum nanoparticles was about a few nanometers in diameter (Fig. 5b). Although XPS analysis revealed that hydrogenase-displaying yeasts produced Pt(0), there is a possibility that black precipitates produced by hydrogenase-displaying yeasts contained other platinum compounds such as PtO or Pt(OH)_2_. Although further analysis is required to demonstrate the composition of the black precipitates, the produced black precipitates could be used as material in fuel cells or catalytic converters.

Platinum was used as a reduction target metal in this study. However, there are some reports that SRB could recover Au(III) as Au (0), in addition to Pt(II) (Creamer et al. 2006). If hydrogenases of SRB contribute to the reduction of Au(III), hydrogenase-displaying yeasts also could reduce Au(III). Furthermore, there are five kinds of metals bearing chemical and physical properties similar to those of platinum [ruthenium (Ru), rhodium (Rh), palladium (Pd), osmium (Os), and iridium (Ir)]. These five metals and platinum are together called platinum group metals (PGMs), and are important because of their catalytic properties (Kettler 2003). Owing to the similarity in their properties, hydrogenase-displaying yeasts could reduce these metal ions. Therefore, the constructed hydrogenase-displaying yeasts could be available as an environmentally friendly technology for the recovery of platinum group metal ions.

## Abbreviations

ICP-MS: inductively coupled plasma-mass spectrometry
PGM: platinum group metal
SRB: sulfate-reducing bacteria
TEM: transmission electron microscopy
XPS: X-ray photoelectron spectroscopy
